# Expert camouflage-breakers can accurately localize search targets

**DOI:** 10.1186/s41235-021-00290-5

**Published:** 2021-04-06

**Authors:** Fallon Branch, Allison JoAnna Lewis, Isabella Noel Santana, Jay Hegdé

**Affiliations:** Department of Neuroscience and Regenerative Medicine, Medical College of Georgia, Augusta University, CA-2003, 1469 Laney Walker Blvd, Augusta, GA 30912-2697 USA

**Keywords:** Accuracy, Categorization, Pop-out, Precision, Visual search

## Abstract

Camouflage-breaking is a special case of visual search where an object of interest, or target, can be hard to distinguish from the background even when in plain view. We have previously shown that naive, non-professional subjects can be trained using a deep learning paradigm to accurately perform a camouflage-breaking task in which they report whether or not a given camouflage scene contains a target. But it remains unclear whether such expert subjects can actually detect the target in this task, or just vaguely sense that the two classes of images are somehow different, without being able to find the target per se. Here, we show that when subjects break camouflage, they can also localize the camouflaged target accurately, even though they had received no specific training in localizing the target. The localization was significantly accurate when the subjects viewed the scene as briefly as 50 ms, but more so when the subjects were able to freely view the scenes. The accuracy and precision of target localization by expert subjects in the camouflage-breaking task were statistically indistinguishable from the accuracy and precision of target localization by naive subjects during a conventional visual search where the target ‘pops out’, i.e., is readily visible to the untrained eye. Together, these results indicate that when expert camouflage-breakers detect a camouflaged target, they can also localize it accurately.

## Significance

In order to recognize a foreground visual object of interest, or target, camouflaged against its background, the viewer must be able to perceptually segregate it from the rest of the image. By definition, effectively camouflaged objects are hard to detect.

We have previously demonstrated the somewhat counterintuitive fact that ordinary, naive subjects can learn to accurately recognize the camouflaged *target* by learning the statistical properties of the *background* (Chen & Hegdé, 2012a). Briefly, subjects were trained using a deep learning task in which they were shown individual camouflage scenes and were required to report whether or not the image contained a target object. Subjects were provided feedback after their response, which served to implicitly and retroactively label the image as one in which the target was present or absent. Importantly, subjects were not told what to learn or what the target object was, nor shown the target in isolation (Chen & Hegdé, 2012b; Streeb et al., 2012). Over several hundred trials (depending on the subject), the subjects’ performance improved to highly significant levels. This occurred regardless of the target object and even when the subjects were never shown the same image twice, so that the only way the subjects could learn the task was by learning the statistical properties of the background, i.e., what a given background ‘looked like,’ so that they could tell when the image contained an ‘odd-man-out’ object that did not share the statistics of the background.

Collectively, these observations suggest that, in order to successfully perform this task, the trained subjects distinguish the overall statistical properties of the images that do not contain a target from the properties of the images that do contain a target. But they also raise an important follow-up question: When such trained experts perform this task, do they detect the actual target, or simply sense that the two classes of images are somehow different? This issue is highly significant in real-world situations. For instance, our finding that naive subjects, regardless of whether or not they have *any *a priori aptitude for visual pattern recognition, can be trained to detect camouflaged targets even upon a very brief viewing (Chen & Hegdé, 2012a); also see (Chen & Hegdé, 2012b; Streeb et al., 2012) is potentially applicable to real-world combat situations. However, merely being able to judge, no matter how accurately, that the given combat scene contains a target is not very useful to a sniper under real-world combat conditions if he/she is unable to also accurately tell *where* the target is.

The localization issue is also significant from a more purely scientific point of view, especially given the fact that expert subjects perform our camouflage-breaking task accurately even when there is no physical target to localize, i.e., when the scene did not contain a physical target object, but simply had the overall statistical properties of images that did [see Fig. 4 of (Chen & Hegdé, 2012a); also see (Chen & Hegdé, 2012b; Streeb et al., 2012)]. Similar findings have been reported in breast cancer screening, where expert radiologists can accurately distinguish mammograms with versus without a lesion even when there is no visible lesion to localize (Brennan et al., 2018; Evans et al., 2016). Thus, it would seem that, at least in principle, localization is dissociable from detection; the latter can occur without the former. But it is unclear whether the ability to localize the target has to be learned separately, or whether it develops as a matter of course when subjects acquire the underlying pattern recognition expertise. Besides, there is evidence that the localization performance is not all that dissociable from detection performance in expert radiologists (Carrigan et al., , 2018, 2019).

We therefore sought to empirically measure the localization performance in expert subjects who had been trained in the camouflage-breaking task, but had received no training whatsoever in localization per se. We show both that when expert camouflage-breakers detect a camouflaged target, they can also localize it quite accurately (Experiment 1). Moreover, this localization performance is statistically indistinguishable from the target localization performance of naive observers in a classical pop-out visual search task (Experiment 2).

## Experiment 1: Localization of camouflaged targets by expert subjects

### Methods

#### Subjects

All procedures used in this study were reviewed and approved in advance by the Institutional Review Board (IRB) of Augusta University in Augusta, GA, where this study was carried out. All subjects were adult volunteers who had normal or corrected-to-normal vision and provided written informed consent prior to participating in the study. All consenting subjects were enrolled in the study; no other inclusion or exclusion criteria were used.

Six subjects participated in Experiment 1. Prior to their participation in this experiment, subjects were trained to criterion using our previously described deep learning method to break camouflage (Chen & Hegdé, 2012a). It is important to emphasize that what the subjects were trained in was a *detection* task, i.e., one in which they had to report whether or not a given image contained an unspecified camouflaged target (i.e., an object that ‘did not belong’). The subjects received no specific training in, nor information, instructions, or feedback about, *localizing* the target. All of the subjects had reached an asymptotic camouflage-breaking performance of *d*′ ≥ 1.95 (*p* < 0.05) during this ‘offline’ training prior to their participation in Experiment 1.

During Experiment 1, each subject performed 4 to 6 blocks (depending on the subject) of 40 trials each. Prior to the actual data collection, the task paradigm was explained to the subjects with the help of figures that pictorially illustrated each step of a typical trial, as well as the organization of the trial blocks and testing sessions. We gave the subjects ample opportunity to ask questions and verbally ascertained that they understood the task. Subjects performed practice trials [mean 4.17 ± 0.75 (SD)] to thoroughly familiarize themselves with the task paradigm before the actual experiment began. The data from the practice trials were discarded.

#### Stimuli

**Fig. 1:**
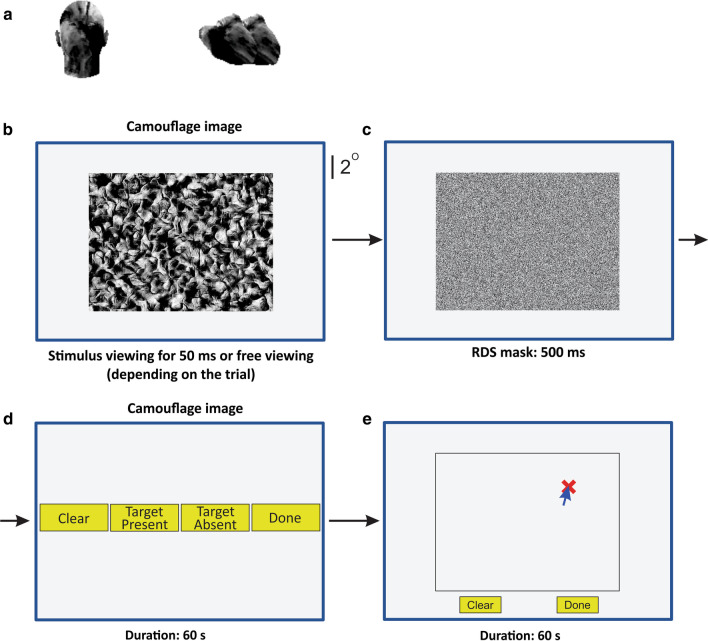
Experiment 1: Visual search for camouflaged target. **a** Target objects. When a target was present, it was either a human head (*left*) or a digital embryo (*right*), each shown here at 4 × their size in actual camouflage images. The human head target can be seen at the top right corner of the camouflage image in panel B. **b**–**e** Task paradigm. Subjects viewed the camouflaged scene (*panel B*), followed by a 0.5 s random dot stimulus mask (RDS mask; *panel C*). Subjects reported whether or not the preceding stimulus contained a target using designated onscreen buttons (*panel D*). Subjects were next presented with a blank outline of the image and were required to report the perceived location of the target (if the target was present) or the center of the image (if the target was absent) using a mouse click. An ‘X’ appeared at the clicked location (*panel E*). Not drawn to exact scale. See text for details

The stimuli used in this experiment were generated as previously described in detail (Chen & Hegdé, 2012a). Briefly, we digitally synthesized a large number of camouflage scenes using (depending on the image) one of three types of naturalistic background textures [“fruit” (see Fig. 1), “foliage,” or “nuts” (see, e.g., Figs. 1 and 4 of (Chen & Hegdé, 2012a))].

A given scene had a 50% chance of containing a single target and 50% chance of containing no target at all. When the stimulus did contain a target, the target had 50% chance each of being a human head (see, e.g., Fig. 1a, *left*) or a novel, naturalistic 3D object called a “digital embryo” [see, e.g., Fig. 1a, *right*; also see (Chen & Hegdé, 2012a; Hauffen et al., 2012]. To determine the pixel location of the target, we divided the image into an imaginary 8 × 6 grid, so that each cell of the grid was 2$^\circ$ × 2$^\circ$. The target was centered in a random location within a randomly selected cell of the grid. We also varied the size of the target at three different scales, so that the longest orthonormal aspect of the target was ~ 0.1$^\circ$, 0.75$^\circ$, or 0.5$^\circ$, depending randomly on the image. Moreover, the target was rotated randomly about its *y*-axis from − 90$^\circ$ to + 90$^\circ$ depending on the image [see (Chen & Hegdé, 2012a) for additional technical details].

The images used in this experiment belonged to the same texture and target class as that in which the given subject was trained. However, no image used during the training was re-used in this experiment, so that the images used during the training versus this experiment constituted two different, non-overlapping subsets of images randomly drawn from the same superset of stimuli.

#### Procedure

Each trial began when the subject fixated on a central fixation spot and indicated readiness by pressing a key on the computer’s keyboard. A single 16$^\circ$ × 12$^\circ$ camouflage scene was then presented as shown in Fig. 1b in one of the following two conditions randomly interleaved in equal proportions within each trial block: (1) free viewing, or (2) time-limited (50 ms) viewing. In either condition, the subjects were able to end the stimulus presentation and proceed to the next phase of the trial by pressing a key. Depending on the trial, the stimulus location was randomly jittered by up to 1$^\circ$. The camouflage scene was followed by a random dot stimulus (RDS) mask presented for 0.5 s (Fig. 1c).

As its name indicates, the RDS consisted of a random field of pixels, each of which had a 50% probability of being black or white.

After the mask was turned off, the subjects reported whether or not the preceding stimulus contained a target using on-screen buttons (Fig. 1, *middle*) and then used a mouse-click to report the perceived center of the target (if present) or the perceived center of the image (if the target was absent). To help determine if the localization performance was affected by whether the subjects had already reported their decision as to the presence or absence of the target, we swapped the order of the two reporting stages of the trial (denoted by panels D and E in Fig. 1) in two blocks each for three subjects (not shown). The data from these trial blocks were indistinguishable from the data with the original trial configuration (data not shown). The two sets of data were therefore pooled.

Data were analyzed using scripts custom-written for *R* (r-project.org) or Matlab (Mathworks.com) platforms. Statistical tests for the accuracy of target localization were carried out using Hotelling’s *T*^2^ test in *R*. Correction for multiple comparisons was carried out using the false discovery rate (FDR) method (Benjamini & Hochberg, 1995).

## Results and discussion

### Expert subjects can accurately localize camouflaged targets

#### Detection performance

As expected, the subjects were able to accurately detect the camouflaged target during the free-viewing condition [mean *d*′ = 2.39 ± 0.29 (SD); *p* < 0.05 overall and for each subject]. The average reaction time was 598 ms ± 96. As also expected, during the 50 ms viewing, the detection performance was slightly lower, albeit still highly significant (mean *d*′ = 2.22 ± 0.41; *p* < 0.05 overall and for each subject). The difference in the detection performance between the two conditions was statistically insignificant (Wilcoxon signed rank test; *V* = 7, *p* > 0.05).

The reaction times during this condition were slightly lower (589 ms ± 103), although statistically indistinguishable from those during the free-viewing condition (Welch Two Sample *t *test; *t* = − 1.55, *df* = 1162.2, *p* > 0.05). This result also held when the reaction times were re-analyzed to take target status (i.e*.,* present/absent) into account using a two-way ANOVA (condition x target status; *p* > 0.05 for both factors and their interaction; also see Table 1, *top row*). Together, the above results indicate that the differences in the detection accuracy between the two conditions were not attributable to a speed-accuracy tradeoff (Luce, 1991).

**Table 1:** Measures of the precision of the localizations in Experiment 1

	Target present		Target absent	
	Free viewing	Stimulus duration: 50 ms	Free viewing	Stimulus duration: 50 ms
Mean reaction time (ms) ± SD	595 ± 95	586 ± 103	604 ± 97	593 ± 103
Mean localization distance ± SD^a^	1.36 ± 0.68	1.44 ± 0.71	1.38 ± 0.73	1.45 ± 0.74

^a^Distance was calculated as the Euclidean distance (in degrees of arc) between the reported location of the target and its actual location during each trial

#### Localization performance

To determine whether the reported locations differed significantly from the actual location, we determined the reported locations across all subjects and trials. Since the actual location of the target varied randomly from one image to the next (see “Methods”), a principled method of comparing the localizations across all trials is to measure the subjects’ reported localization during each given trial as the deviation from the actual physical location of the target during that trial.

**Fig. 2:**
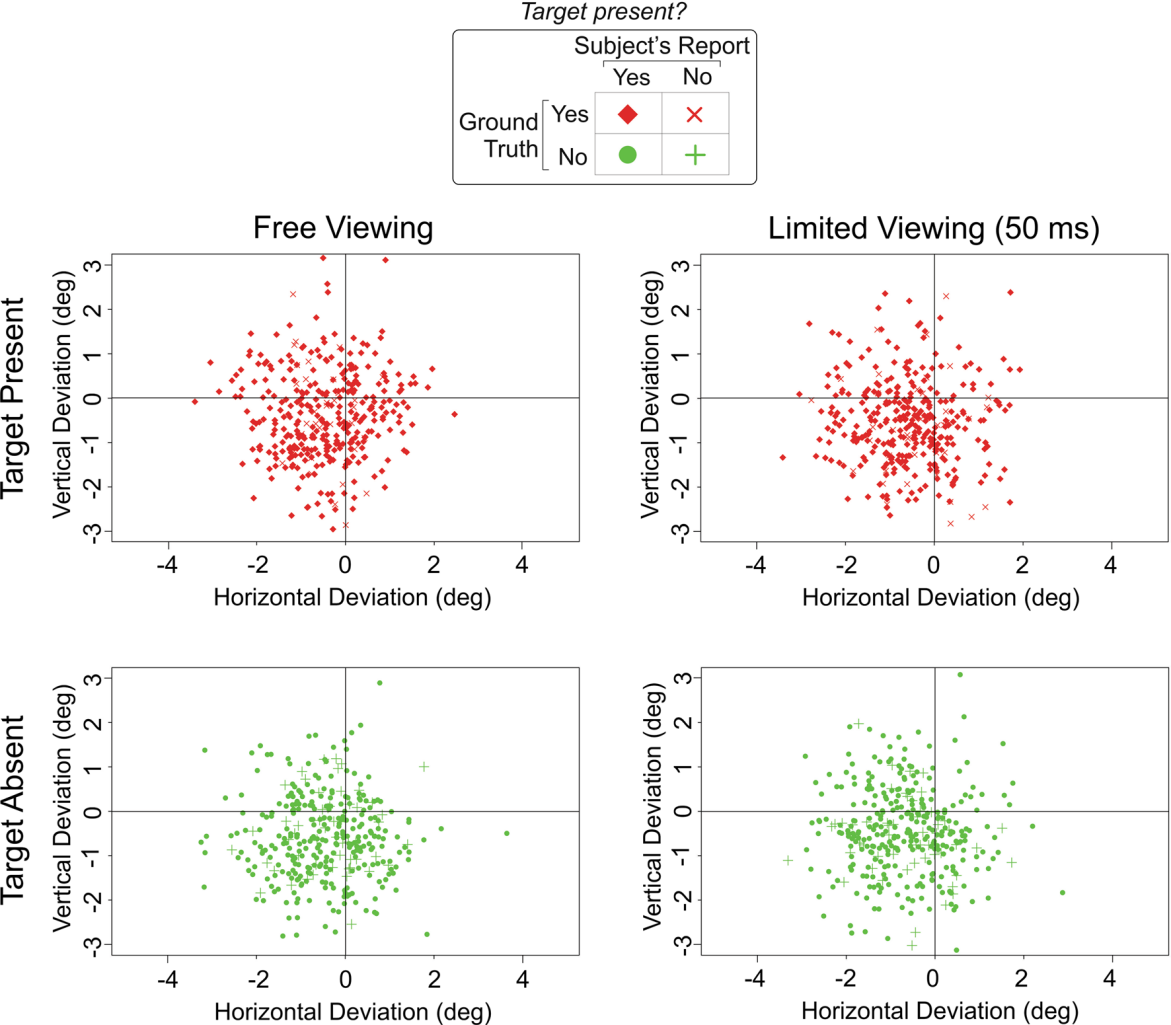
Localization of camouflage targets in Experiment 1. The scatter plots in the *left column* show the localization data for free viewing. Scatter plots in the right column show the data for time-limited viewing of 50 ms. Scatterplots in the *top* and *bottom rows* show data for stimuli with or without a target, respectively. Scatterplots in the *left* and *right columns* show data for stimuli viewed freely or for 50 ms, respectively. Each *plotting symbol* in each scatterplot denotes the localization data from a single trial (see *inset* at *top center*), plotted as the angular deviation of the reported target location from the actual location, so that the cross-hairs denote perfectly accurate localization. Note that a slight skew of the localization data toward the lower left was visually apparent in each panel. However, this was not statistically significant (not shown). The likeliest cause of this skew is the difference between the perceived center of the target object versus the nominal physical center of the object (not shown)

The top left panel of Fig. 2 shows the results for freely viewed stimuli with targets. The degree to which the reported location deviates, or is separated, from the actual location of the target (denoted by the *crosshair*) constitutes the accuracy of localization (Dodge, 2003; Green & Swets, 1966; Van Trees, 2001), so that a perfectly localized target would be denoted by a plotting symbol centered on the crosshair. The spread (or, in statistical terms, variance) of the reported locations from the actual location constitutes its precision (Dodge, 2003; Green & Swets, 1966; Van Trees, 2001).

To determine whether the reported locations differed significantly from the actual location, we used a Hotelling’s *T*^2^ test, which, like many statistical tests of significance, balances the two-dimensional [2D] separation (i.e., accuracy) versus spread (i.e., precision) (Henkel, 1976; Hotelling, 1931). We used it to test the alternative hypothesis that the reported 2D target locations deviated significantly from the actual target’s 2D location (*μ* = {0,0}, denoted by the crosshair). We found that when the subjects were able to view the camouflage scene freely (Fig. 2, *top left*), the reported locations of the targets (*red symbols*) were statistically indistinguishable from the actual location of the target (*center* of *crosshair*; Hotelling’s *T*^2^ test, *F*(1,700) = 0.76, *p* > 0.05, FDR-corrected for multiple comparisons). Thus, when the subjects had ample opportunity to view the camouflage image, their reported target locations were non-random and were centered on the actual location of the target instead.

To help determine whether and to what extent the stimulus duration per se affected the accuracy of target localization, we examined the accuracy of localization for the stimulus duration of 50 ms. Note that a stimulus duration this brief allows for little in the way of eye movements (Ibbotson & Krekelberg, 2011; Kowler, 2011) [also see (Hess et al., 2016)]. Nonetheless, subjects were able to localize the target in this case as well (Fig. 2, *top right*; Hotelling’s *T*^2^ test, *F*(1,700) = 0.07, *p* > 0.05, FDR-corrected). Indeed, target localization after 50 ms viewing was statistically indistinguishable from localization after free viewing (Hotelling’s *T*^2^ test, *F*(1,1398) = 6.41, *p* > 0.05, corrected). That is, the subjects were able to localize the targets accurately even upon a brief viewing of the stimulus.

As noted in the Methods section, when the stimulus did not contain a target, subjects were instructed to report the location as the center of the stimulus. This provided a baseline measure for how accurate the subjects were in localizing the target. We found that the localization in these cases was statistically indistinguishable from localization when the target was present (Hotelling’s *T*^2^ tests, *p* > 0.05, corrected).

Together, the above results show that subjects were, in a statistical manner of speaking, highly accurate in their localizations. But the localizations were self-evidently imprecise; the reported target locations deviated substantially from the actual target locations (see Fig. 2). Summary statistics shown in Table 1 (*bottom row*) indicate that the subjects misestimated the target location by about 1.45° on average. The precision of target localization did not vary as a function of stimulus duration and of whether the stimulus contained a target (two-way ANOVA, stimulus duration × target status, *p* > 0.05 for both factors and their interaction). This straightforwardly indicates that these factors were not the main source of the localization errors (see General Discussion below).

## Experiment 2. Localization of pop-out targets by naive subjects

In this experiment, the stimuli consisted of conventional visual search arrays that are known to elicit perceptual ‘pop-out’ (Treisman, 1988; Wolfe, 1994). The rationale for using pop-out search arrays was that expert camouflage-breakers report that the subjective experience of searching for a camouflaged target is similar to searching for a pop-out target in a conventional visual search array (Chen & Hegdé, 2012b), in that the target is effortlessly visible.

**Fig. 3:**
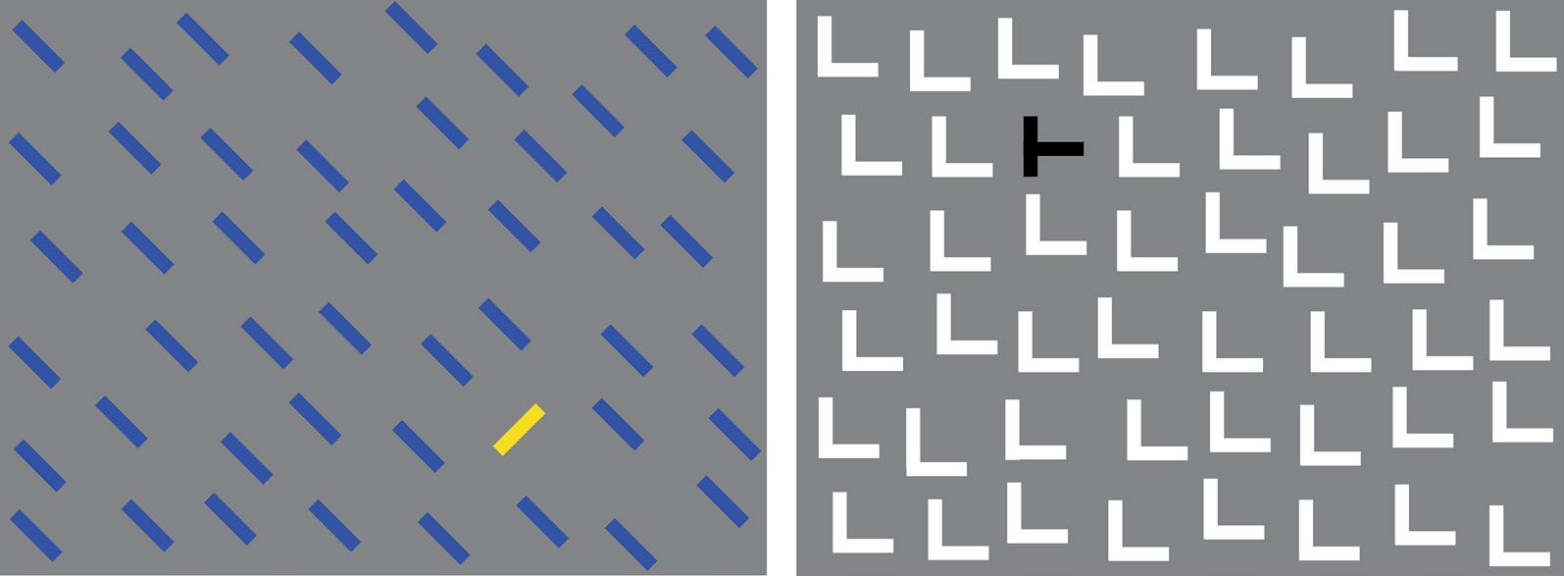
Exemplar conventional visual search stimuli used in Experiment 2. See text for details

This experiment tested the hypothesis that the localization performance observed in Experiment 1 was not idiosyncratic to the camouflage-breaking task nor unusually imprecise, but was comparable to the target localization performance in other visual search tasks. In this experiment, subjects performed the same task as in Experiment 1, but used visual search arrays where the target was readily recognizable (or ‘popped-out’; see Methods for details; also see Fig. 3).

### Methods

#### Subjects

Subject were recruited and consented exactly as in Experiment 1, except that seven subjects who did not participate in Experiment 1 participated in this experiment.

#### Stimuli

In this experiment, instead of camouflage scenes, conventional pop-out visual search arrays were used as stimuli. Since Experiment 1 featured six different types of stimuli (2 targets × 3 background types), we used six different types of pop-out stimuli in this experiment: yellow 45° bar target (when present) among blue 135° bar distractors (Fig. 3a), black T-shaped target among white L-shaped distractors (Fig. 3b), yellow vertical bar target among yellow horizontal bar distractors (not shown), white Q-shaped target among white O-shaped distractors (not shown), black O-shaped target among black C-shaped distractors (not shown), and blue S-shaped target among green H-shaped distractors(not shown). Each of these stimuli has been previously shown to result in perceptual pop-out ((Treisman, 1998); also see (Treisman, 1988; Wolfe, 1994)).

To help make the target location in this experiment analogous to the target location in Experiment 1, the arrays were created by dividing the stimulus into the aforementioned 8 × 6 grid, so that in the stimuli without a target, there were 48 distractors, one in each cell of the grid. The location of each distractor within its cell was randomly jittered by up to 0.35° in a random direction. In the remaining stimuli, there was a single target that ‘popped out,’ or was readily recognizable, located in a randomly selected cell of the grid, accompanied by 47 distractors in the remaining cells.

#### Procedure

The procedure was identical to that used in Experiment 1, except in the following respects: In this experiment, the aforementioned pop-out stimuli were used instead of camouflage images. Before the actual experiment, subjects carried out an average of 5.29 ± 1.80 practice trials. Subjects received no other training or practice of any kind.

## Results and discussion

### Detection performance

The detection performance in this experiment was statistically indistinguishable from that in Experiment 1 using a three-way ANOVA (experiment × condition × target status; *p* > 0.05 for all factors). Specifically, the subjects were able to accurately detect the pop-out target during the free-viewing condition [mean *d*′ = 2.27 ± 0.29 (SD); *p* < 0.05 overall and for each subject]. The average reaction time was 634 ms ± 95. As also expected, during the 50 ms viewing, the detection performance was slightly lower, albeit still highly significant (mean *d*′ = 2.11 ± 0.35; *p* < 0.05 overall and for each subject). The difference in the detection performance between the two conditions was statistically insignificant (Wilcoxon signed rank test; *V* = 5, *p* > 0.05).

The reaction times during this condition (529 ms ± 95) were statistically indistinguishable from those during the free-viewing condition (Welch Two Sample *t *test; *t* = − 0.96, *df* = 1241.1, *p* > 0.05). This result also held when the reaction times were re-analyzed to take target status (i.e*.,* present/absent) into account using a two-way ANOVA (condition × target status; *p* > 0.05 for both factors and their interaction; also see Table 2, *top row*). Together, the above results indicate that the differences in the detection accuracy between the two conditions were not attributable to a speed-accuracy tradeoff in this experiment either.

**Table 2:** Measures of the precision of the localizations in Experiment 2

	Target present		Target absent	
	Free viewing	Stimulus duration: 50 ms	Free viewing	Stimulus duration: 50 ms
Mean reaction time (ms) ± SD	637 ± 95	631 ± 100	627 ± 96	626 ± 95
Mean localization distance ± SD^a^	1.38 ± 0.70	1.52 ± 0.78	1.43 ± 0.70	1.45 ± 0.73

^a^Distance was calculated as the Euclidean distance (in degrees of arc) between the reported location of the target and its actual location during each trial

Localization performance during the pop-out visual search is comparable to the localization performance in camouflage-breaking tasks.

**Fig. 4:**
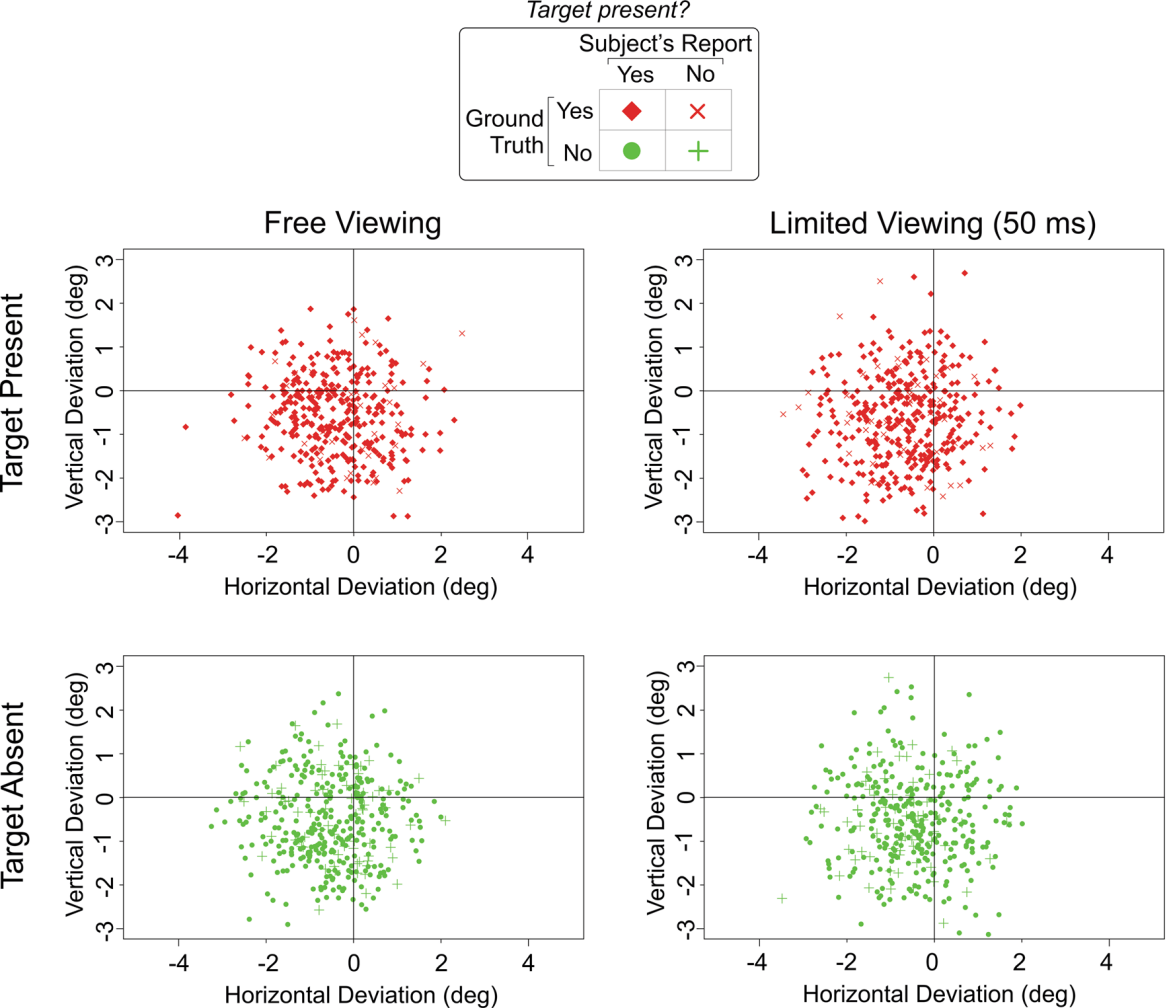
Localization of camouflage targets in Experiment 2. Plotting conventions are the same as in Fig. 2. See text and legend to Fig. 2 for details

We found that the results from this experiment were qualitatively similar to the results from Experiment 1 (Fig. 4 and Table 2). To quantitatively compare the results between the two experiments, we carried out a three-way analysis of variance (ANOVA) using three factors with two levels each: Experiment (1 vs. 2) × condition (free-viewing vs. 50 ms) × target status (present vs. absent). We found that none of the factors or their interactions had a statistically significant effect on localization performance (*p* > 0.05 in each case). This result confirms the hypothesis that the localization performance Experiment 1 was not idiosyncratic to the camouflage-breaking task nor unusually imprecise, but was comparable to the target localization performance in the conventional parallel visual search (pop-out) tested in Experiment 2.

## General discussion

Our results show that expert subjects can accurately tell where the camouflaged target is in a camouflage scene, and they can do so even upon viewing the stimulus as briefly as 50 ms. This localization performance was statistically indistinguishable from the performance of naive subjects in a conventional pop-out search task. That is, trained camouflage-breakers can find a target in a camouflage scene as quickly and accurately as untrained subjects can find a target that pops-out of a conventional visual search array.

If the subjects were performing the task based on the perceived differences in the overall statistical properties of images with versus without a target, they would be able to localize the center of the image when the target was absent, because one can, in principle, do this by visually gauging the center of the image outline provided during the localization phase of the trial (Fig. 1, *right panel*). However, they would not be able to accurately localize the *actual target* using this strategy. Therefore, in this scenario, the localization performance can be expected to be better when the target was absent versus localization when the target was present. However, this was not what we empirically observed; the localization was statistically indistinguishable between the two cases, but accurate in both cases. Together, these considerations suggest that the subjects actually perceived the physical target when it was present. This was also consistent with the subjective percepts verbally reported by the subjects after their participation in the study was completed (not shown).

These findings have important potential applicability to real-world combat situations because they imply, taken together with the fact that the expert subjects were not trained specifically in the localization task, that expert camouflage-breakers do not have to be specifically trained in the localization task. The localization expertise evidently develops as a matter of course of acquiring the target detection expertise using our deep learning paradigm. This raises additional intriguing questions as to whether this is idiosyncratic to our training paradigm, and whether and to what extent localization versus detection expertise develop concurrently during the training. Additional studies are needed to resolve these issues.

As noted above, while the expert camouflage-breakers were able to localize the camouflaged target as accurately as naive subjects localized targets that popped out, neither localization performance was not all that precise. After all, in real-world combat situations, a sniper who localizes the target with a precision of about 1.45° of arc leaves much to be desired. It is possible that the poor precision is somehow an inherent limitation of our deep learning training protocol. However, this is unlikely to be the sole cause of the imprecision, especially in view of the fact naive subjects who received no training performed just as imprecisely using a substantially different type of stimuli. It is possible the errors are attributable, at least in part, to random and/or systematic motor errors [see, e.g., (Pelisson & Prablanc, 2009)] and the fact that the target themselves varied in size from 0.5° to 1°, depending on the trial. It is also worth noting that in both experiments, the stimulus had been turned off and masked by the time subjects got an opportunity to localize it. That is, our subjects localized a *remembered* target, and not a *visible* one. Previous studies using other sensorimotor tasks have shown that the neural information about target location decays, and the magnitude of localization errors increases, rapidly over time, especially in the absence of a visible localization target [see, e.g., (Binder et al., 2009; Pelisson & Prablanc, 2009)]. Thus, it is plausible that similar temporal decay of the location information about the target contributed, in part, to the localization errors. A related issue is whether the precision of localization is different in higher-level categorization tasks, e.g., when the subjects have to determine if the human head is that of a friend or foe, compared to the low-level detection (*target present or not?*) task used in the present study. These and the aforementioned issues raised by the present preliminary study point to some useful future directions of research.

## Data Availability

The images used in the study and the deidentified data are available for non-commercial use upon reasonable request.
